# Effects of Invasive-Plant Management on Nitrogen-Removal Services in Freshwater Tidal Marshes

**DOI:** 10.1371/journal.pone.0149813

**Published:** 2016-02-25

**Authors:** Mary Alldred, Stephen B. Baines, Stuart Findlay

**Affiliations:** 1 Department of Ecology and Evolution, Stony Brook University, Stony Brook, New York, United States of America; 2 Cary Institute of Ecosystem Studies, Millbrook, New York, United States of America; Shandong University, CHINA

## Abstract

Establishing relationships between biodiversity and ecosystem function is an ongoing endeavor in contemporary ecosystem and community ecology, with important practical implications for conservation and the maintenance of ecosystem services. Removal of invasive plant species to conserve native diversity is a common management objective in many ecosystems, including wetlands. However, substantial changes in plant community composition have the potential to alter sediment characteristics and ecosystem services, including permanent removal of nitrogen from these systems via microbial denitrification. A balanced assessment of costs associated with keeping and removing invasive plants is needed to manage simultaneously for biodiversity and pollution targets. We monitored small-scale removals of *Phragmites australis* over four years to determine their effects on potential denitrification rates relative to three untreated *Phragmites* sites and adjacent sites dominated by native *Typha angustifolia*. Sediment ammonium increased following the removal of vegetation from treated sites, likely as a result of decreases in both plant uptake and nitrification. Denitrification potentials were lower in removal sites relative to untreated *Phragmites* sites, a pattern that persisted at least two years following removal as native plant species began to re-colonize treated sites. These results suggest the potential for a trade-off between invasive-plant management and nitrogen-removal services. A balanced assessment of costs associated with keeping versus removing invasive plants is needed to adequately manage simultaneously for biodiversity and pollution targets.

## Introduction

Invasive species are often presumed to degrade ecosystem functioning. By causing local extinctions, invasives are thought to reduce biodiversity [1], which is often correlated to ecosystem functions [2]. As many ecosystem functions provide essential services for human survival and well-being, these relationships imply that preservation of biodiversity is important for environmental management [3], particularly when ecosystems provide multiple ecosystem services [2, 4]. Consequently, eradicating invasive species has become a common management practice. That said, invasive species are often better than native species at sequestering limiting resources into biomass [5, 6]. Conflicts between management goals can occur if, after invasion, this difference in ability results in improved nutrient storage or removal at the same time other services are degraded [5]. Quantifying the effect of invaders on nutrient storage and removal processes is necessary to assess the full ecological effect of invasions, and to prioritize the allocation of limited management resources. This tradeoff may be especially important in wetlands. These ecosystems provide a diverse array of ecosystem services, including flood abatement, support for biodiversity, and improvement of water quality. Together these services have been valued at approximately $3000 per hectare annually [7]. At least 50% of the global wetland area has been lost to agricultural, urban, and rural development and much of the remaining wetlands are considered “degraded.” [8, 9].

Wetlands have been found to be particularly susceptible to invasions by opportunistic plant invaders that form monotypic stands and displace native plant species. In fact, over 24% of the "worst plant invaders" are wetland plants [10]. One of the best known of these wetland plant invaders is *Phragmites australis* [11]. Although *Phragmites* has been at least a minor component of brackish marshes in the United States for over 40,000 years [12], cryptic invasions of European haplotypes of *Phragmites* beginning in the 1800s caused a dramatic increase in abundance and size of *Phragmites* stands, as well as aggressive colonization into freshwater and brackish marshes beyond the limits of the native’s historic range [13]. *Phragmites* alters a wide range of wetland ecosystem services. The dense, tall growth of *Phragmites* stands often cause local reductions in the native diversity of plant species following invasions [14–16]. *Phragmites* is also often believed to provide poor habitat for native birds, fish, and invertebrates relative to native wetland plants [17, 18] although it clearly provides habitat for some organisms [19]. For these and aesthetic reasons, management organizations in the United States invest considerable sums, an estimated $4.6 million annually [20], on the control and eradication of *Phragmites*. However, the same traits that allow *Phragmites* to form dense monocultures can also promote ecosystem services such as nutrient and pollutant remediation [21, 22], shoreline stabilization [23], and maintenance of wetland habitat in disturbed and urban areas [19].

Throughout many highly developed and agricultural regions of the United States, excessive nitrogen loading poses a particular challenge to managing local water quality. The invasive haplotypes of *Phragmites* are particularly adept at colonizing disturbed or nutrient-rich aquatic systems [24], and once established reach heights of up to 4 meters and biomass densities of 727–3663 g DM m^-2^ [25]. Consequently, *Phragmites* can sequester much larger pools of nitrogen in biomass than can the native wetland plants it replaces [22]. *Phragmites* also aerates wetland sediments that would otherwise remain anoxic [26], allowing for greater production of nitrate (nitrification) and thus greater removal of nitrogen to the atmosphere as inert dinitrogen gas (via denitrification) [27, 28]. Though a great deal of research has focused on the removal of *Phragmites* through mechanical, chemical, or biological means [29], relatively few studies have quantified the effect of eradication on nitrogen-removal ecosystem services (but see [30, 31]). Moreover, the characteristics of the plant communities that recolonize treated areas are even more infrequently characterized in terms of these services [29].

Our study sought to investigate the impacts of small-scale *Phragmites australis* removals on sediment nutrient concentrations and denitrification potential. Using two years of pre-removal monitoring data and two years of post-removal monitoring data, we compared the nitrogen removal rates of treated sites to intact *Phragmites* stands and sites dominated by native *Typha angustifolia*. We also assessed the species composition of treated sites two years following removal, and compared traits relevant to nitrogen removal between the recolonizing plant community and plant communities at sites dominated by *Phragmites* and *Typha*. We hypothesized that removal of *Phragmites* should lower rates of sediment aeration, thereby decreasing rates of nitrate production from ammonium via nitrification and reducing the supply of nitrate to denitrifying microbes. We further hypothesized that the absence of plant N uptake should further limit removal of ammonium from sediments to plant biomass. Therefore, we predicted that ammonium concentrations should increase in sediments following *Phragmites* removal and that denitrification potentials should decrease relative to vegetated plots. To our knowledge, this is only the second study to investigate the impacts of *Phragmites* removal on sediment denitrification potential [30] and the first to have the opportunity to do so within replicated removal stands.

## Methods

### Site Description

**Fig 1:**
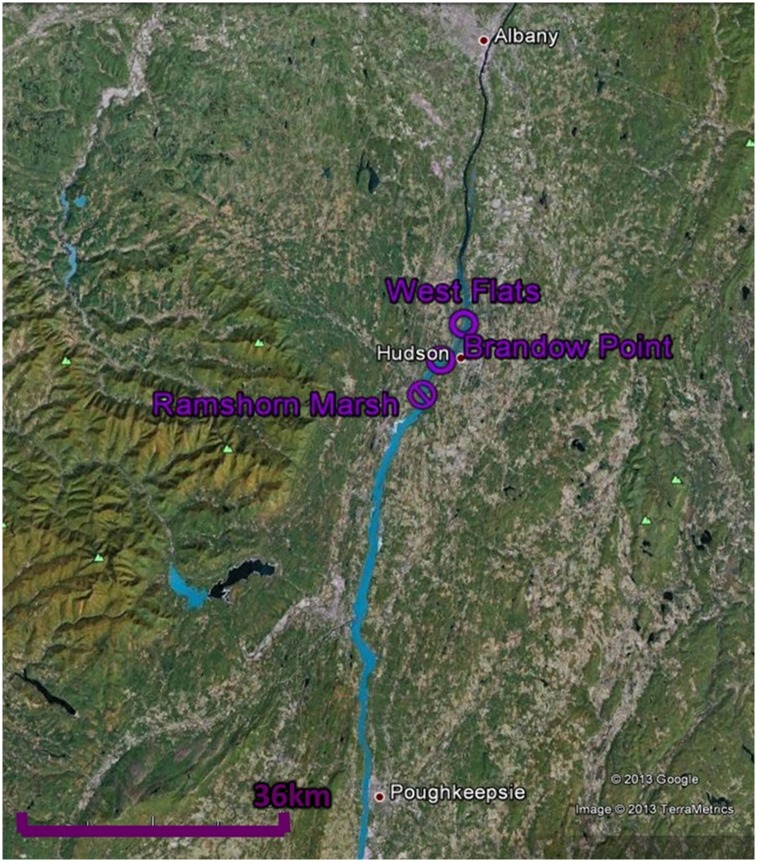
Map of sites included in field sampling. *Phragmites* removals ocurred at Ramshorn Marsh. Intact *Phragmites* stands were monitored at West Flats and Brandow Point (hereafter referred to as “Reference” sites). *Typha*-dominated communities were monitored at all sites.

In September 2010, the Eastern New York (ENY) Chapter of the Nature Conservancy, with permission from Audubon New York and the Scenic Hudson Land Trust, initiated small-scale (0.30–0.76 ha) eradications of three stands of *Phragmites australis* from Ramshorn Marsh (N 42.216059, W -73.854959), a 308 ha freshwater tidal marsh located on the west shore of the Hudson river near Catskill, NY (Fig 1). Initial removals were performed with an application of Aquapro^™^ glyphosate herbicide (7.0 L/ha) and LI-700 aquatic surfactant (1.25 ppm) from a Marsh Master^™^ using a mounted spray system. Subsequent spot treatments of Aquapro^™^ (1%) and LI-700 were applied in September 2011 and 2012 using low-volume backpack sprayers [32].

We conducted an initial survey of denitrification potential, sediment organic content, and sediment ammonium and nitrate concentrations in the three *Phragmites* stands in Ramshorn Marsh in August 2009 prior to removal. Four replicate locations were sampled in each of the stands. In August 2010, we expanded our pre-treatment survey to include three reference stands of *Phragmites*, one at Brandow Point (N 42.249440, W -73.824390) and two at West Flats (N 42.295549, W -73.786796), which were not selected for removal (Fig 1). These sites were similar to Ramshorn in terms of sediment chemistry and hydrology prior to treatment [33], and are located on the west shore of the Hudson River 5.4 km and 11.3 km northeast of Ramshorn Marsh, respectively. In 2010, we performed the same measurements conducted in 2009 at two locations within each *Phragmites* reference stand, one location within each *Phragmites* stand in Ramshorn designated for removal, and one paired *Typha*-dominated area near each Ramshorn *Phragmites* stand (total of 12 sampling locations). Following removal in September 2010, we repeated our measurements in September 2011, June 2012, and September 2012. At each of these times, we sampled three locations in the reference *Phragmites* stands (hereafter “Reference-*Phragmites*”), three locations in the treated *Phragmites* stands (“Ramshorn-Removal”), and six locations in *Typha*-dominated areas near each of the *Phragmites* stands (“Reference-*Typha*” and “Ramshorn-*Typha*”).

### Vegetation

In September 2011 and June 2012, we measured aboveground biomass and leaf carbon and nitrogen content for all locations sampled at the reference sites, but we refrained from collecting biomass samples at Ramshorn due to concerns that *Phragmites* regrowth could escape follow-up herbicide treatments if clipped. In September 2012 we received permission from the Nature Conservancy to add biomass and leaf carbon and nitrogen content measurements to sampling at Ramshorn locations as well. We harvested all aboveground biomass within two haphazardly placed quadrats (25 x 25 cm) within a 2 m radius of each sediment-sampling location. Harvested biomass was dried, weighed, and subsampled for analysis of leaf carbon and nitrogen content using a Perkin Elmer Series II CHNS Analyzer [34]. For September 2012 data, we identified harvested biomass to species and determined dry mass and leaf carbon and nitrogen content for each component plant species within each quadrat. Plot-level data for vegetation measurements were calculated as an average weighted by the biomass of the component species.

### Porewater Nutrients

We measured sediment nutrient profiles using PVC porewater equilibrators with 12 ml sampling wells spaced vertically at 3 cm intervals. Equilibrators were filled with deionized water, covered with a Spectr-Por cellulose membrane, and deoxygenated overnight by bubbling with nitrogen gas prior to installation in sediments [35]. Equilibrators were left in vegetated sediments at each sampling location for 7–10 days. Porewater was collected from sampling wells using a syringe and stored in acidified vials until analysis. In 2009, samples were analyzed for ammonium and nitrate by ion chromatography and in all subsequent years using standard colorimetric methods [36, 37]. After 2010, porewater was also sampled from ~5–10 cm below the sediment surface using a syringe-vacuum porewater sipper. Sipper samples were frozen at -20°C until analysis using standard colorimetric methods.

### Denitrification potential

Duplicate sediment cores from each sampling location were collected with a 7 cm diameter metal corer to a depth of ~10–15 cm for assays of denitrification potential. Samples were stored at 4°C and analyzed within 24 hours of sampling. A five gram subsample was amended with potassium nitrate (KNO_3_), glucose, chloramphenicol, and acetylene and incubated under anaerobic conditions for 90 minutes [38]. Headspace samples were collected at 30 and 90 minutes and stored in pre-evacuated glass vials. Gas samples were analyzed for N_2_O using electron capture gas chromatography. This method provides an indication of the amount of denitrifying enzyme present in the soil and has been demonstrated to be a reliable measurement for comparing the potential for microbial communities to perform denitrification among experimental treatments or sites. While useful for detection of relative changes in denitrification among treatment plots [39], these denitrification potentials should not be interpreted as a measure of absolute denitrification rates [40].

A subsample of each core was also used to determine sediment moisture content as the change in mass after drying at 70°C for a minimum of 24 hr, total organic content as the loss after combustion at 450°C for 4 hr [41], and total nitrogen and carbon content [34].

### Statistical Analysis

Differences in plant community composition among the three dominant vegetation types [*Phragmites*, *Typha*, Removal] were assessed using discriminant analysis of duplicate aboveground-biomass plots in September 2012. This method attempts to characterize an a priori grouping (in this case, three dominant vegetation types), based on a linear combination of predictor variables (in this case, biomass of the component species occupying the plots). If the plant community at the removal sites reverted to a *Typha*- or *Phragmites*-dominated community within the time frame of our study, the discriminant analysis would fail to distinguish the removal sites from one of these dominant vegetation types. If the plant community at removal sites switched to a novel community following herbicide treatment, the discriminant analysis would succeed in assigning removal plots to this novel vegetation type.

Differences in plant traits [aboveground biomass, leaf nitrogen content, total plant nitrogen content] measured in September 2012 among the three dominant vegetation types [*Phragmites*, *Typha*, Removal] were analyzed with a one-way ANOVA. A separate two-way ANOVA was used to compare *Phragmites* and *Typha* communities sampled at the reference sites at two additional sampling times [September 2011, June 2012, September 2012]. Measurements of sediment nitrogen and carbon collected August 2010-September 2012 were also analyzed with a two-way ANOVA to compare the three dominant vegetation groups. For each two-way ANOVA, potential interactions between vegetation type and sampling time were tested for significance.

Measurements of denitrification potential, sediment porewater nutrients, and total sediment organic content were analyzed at the stand scale using two-way ANOVA. Because the “Reference-*Typha*” treatment group was not added until September 2011, it was necessary to analyze the data using two different statistical designs. The first compared the three “treatments” [Ramshorn-Removal, Ramshorn-*Typha*, Reference-*Phragmites*] that were sampled across all “sampling times” [August 2010, September 2011, June 2012, September 2012], both before *and* after herbicide treatment. For these ANOVAs, significant impacts of herbicide treatment on dependent variables [denitrification potential, sediment ammonium content, and sediment organic content] were tested as planned comparisons within the interaction term (treatment x sampling time). In the second statistical design, we compared four treatments [Ramshorn-Removal, Ramshorn-*Typha*, Reference-*Typha*, Reference-*Phragmites*] for sampling times that occurred after herbicide treatment [September 2011, June 2012, September 2012]. For these ANOVAs, significance of herbicide treatment was assessed for the dependent variables listed above and additional measurements added in 2011 [total organic carbon and nitrogen content of sediments] using planned comparisons of treatments.

The sediment properties and processes we measured are notoriously variable as a result of measurement difficulties and spatial variability [40], and sample size was necessarily limited due to the number of treatments conducted. For these reasons, our study was particularly susceptible to Type II error. To limit the likelihood of committing a Type II error, we adopted a higher α value of 0.1. Because the planned contrasts included in the ANOVAs described above are not orthogonal, we applied corrections for multiple comparisons using the false-discovery-rate method described in Benjamini and Hochberg [42]. This method has been shown to control the proportion of falsely rejected null hypotheses without sacrificing statistical power. Such methods are appropriate in cases such as ours when the failure to reject a false null (committing a Type II error) is equally as problematic as rejecting a true null (committing a Type I error) [43].

Planned comparisons of Ramshorn-Removal and Reference-*Phragmites* sampling times were used to test for significant differences between sites where *Phragmites* was removed relative to sites where *Phragmites* was left intact. Planned comparisons of Reference-*Typha* and Reference-*Phragmites* were used to test whether sites dominated by plant species differed when left undisturbed. Though we attempted to control for phenology by sampling at peak biomass in each of our sampling years (late August/early September), we acknowledge that other factors such as temperature may influence sediment chemistry and microbial activity. The sampling conducted in June 2012 was added to provide further insight into the short-term trajectory of sediment recovery in treated sites. Planned comparisons of treatment and reference sites statistically control for interannual and seasonal variability when detecting treatment effects. All statistical analyses were performed in JMP [44]. Graphs were produced in R using the package ggplot2 [45]. Complete results of all statistical comparisons, false-discovery-rate corrections, and code used to generate figures are available in supporting information.

## Results

### Vegetation

**Fig 2:**
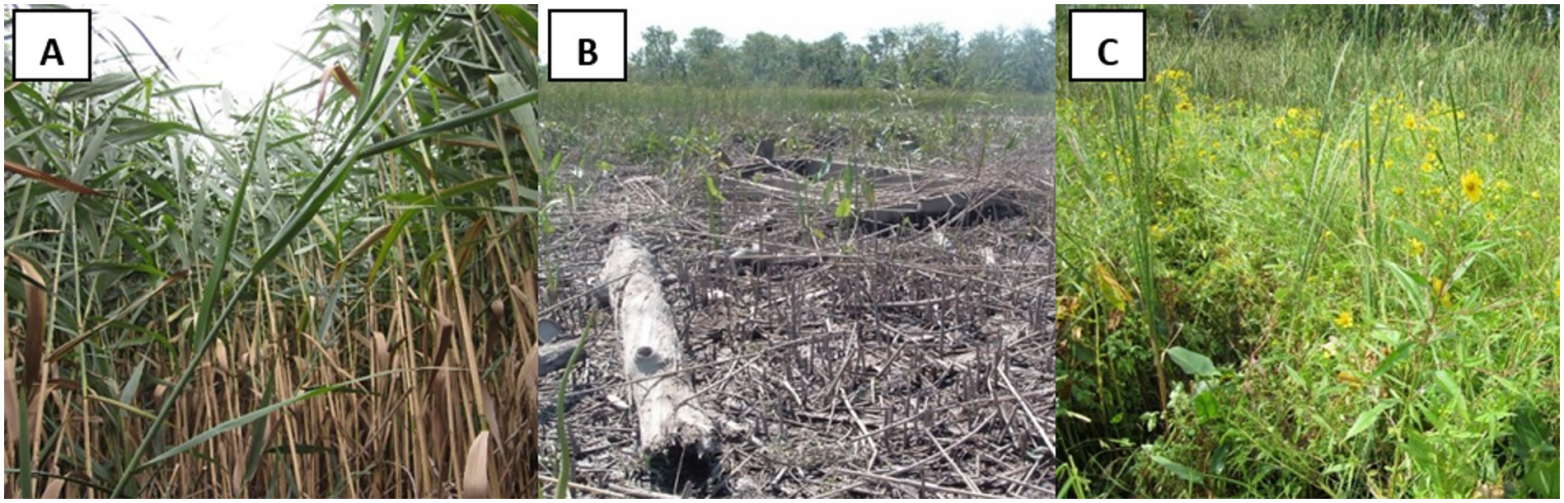
Photos from Ramshorn Marsh. Photos were taken (A) prior to removal in August 2010, (B) one year following removal in September 2011, and (C) two years following removal in September 2012.

**Fig 3:**
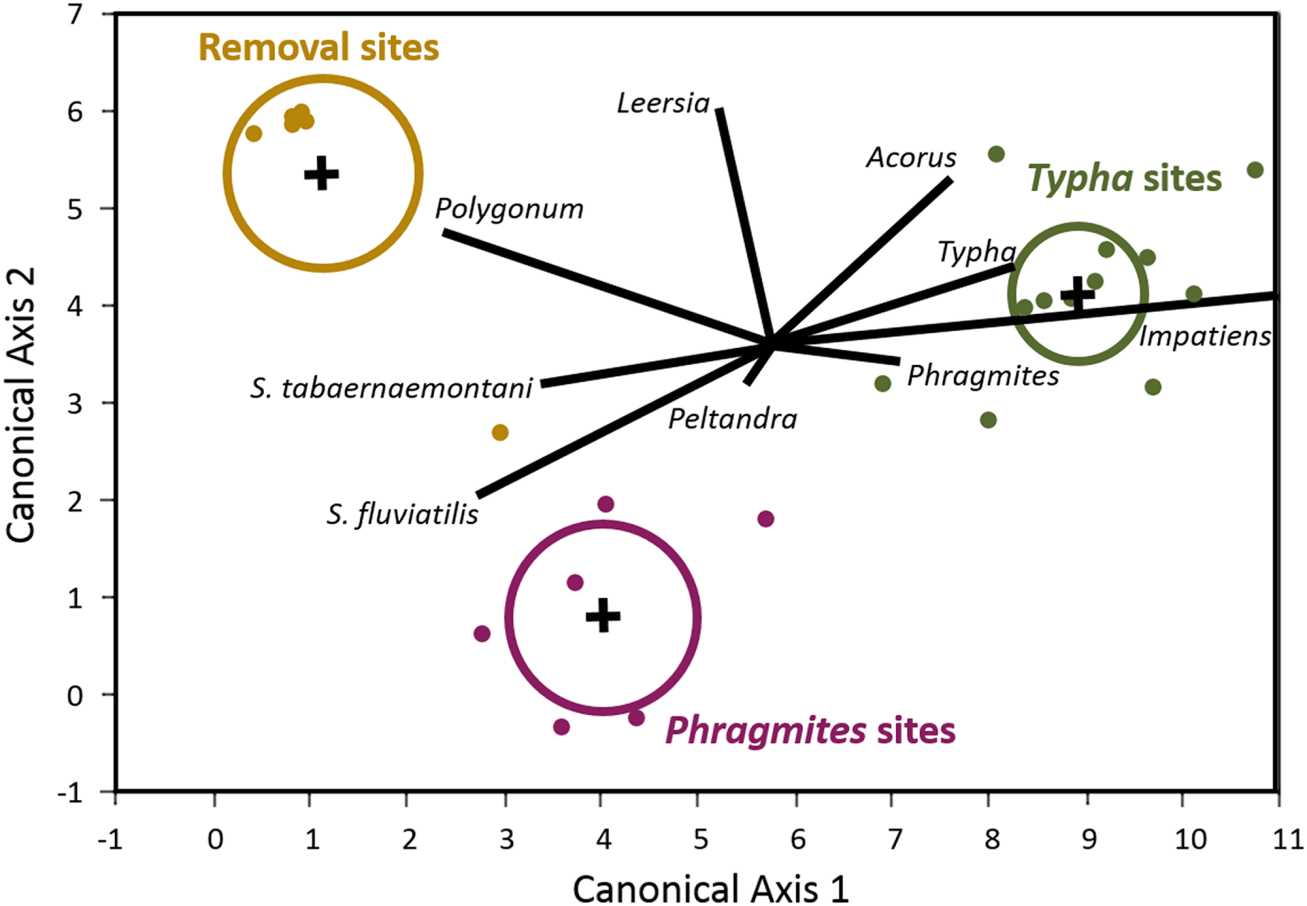
Biplot of dicriminant analysis for three dominant vegetation classifications [*Phragmites*, *Typha*, Removal] sampled in September 2012. Independent variables used to discriminate among the classifications were the biomasses of the component plant species at individual sampling plots. Rather than reverting from a *Phragmites*-dominated (purple) to a typical *Typha*-dominated community (green), communities shifted to a novel community characterized by *Leersia oryzoides* and *Polygonum arifolium* (brown) two years after initial herbicide treatment.

**Fig 4:**
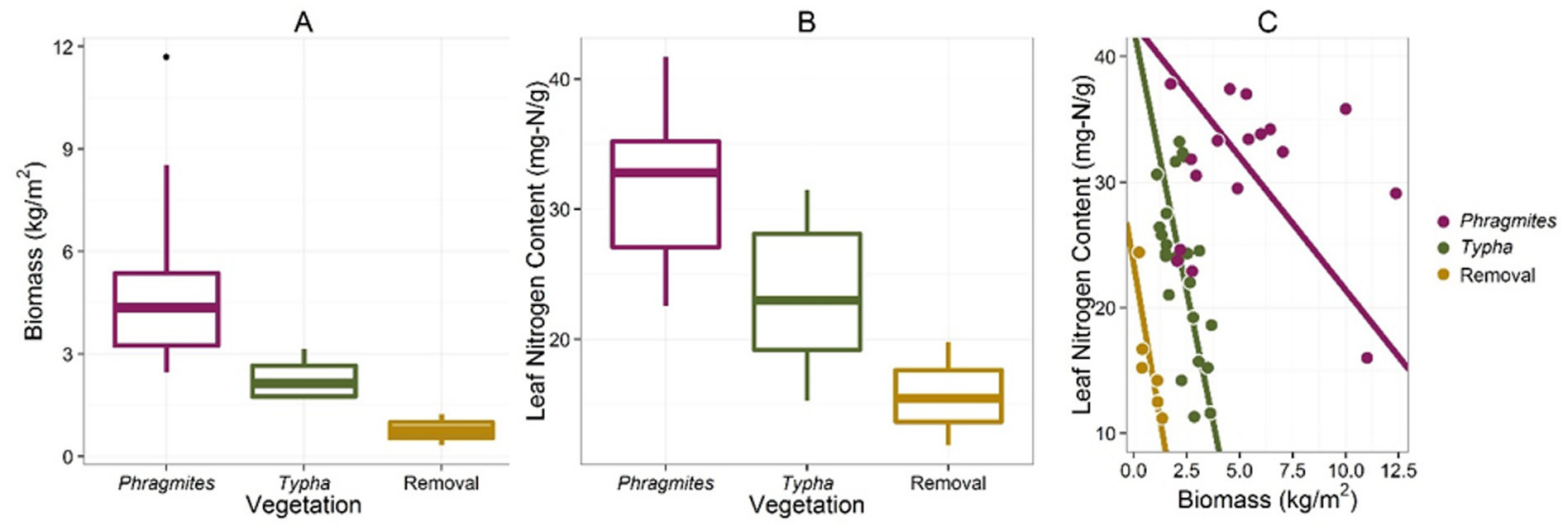
Differences in (A) aboveground biomass and (B) leaf nitrogen content among the three dominant vegetation classifications. *Phragmites*-dominated communities reach a higher peak biomass and assimilate more nitrogen per gram of leaf material than *Typha*-dominated and removal communities. Boxplots represent the median and interquartile range, and whiskers extend to the most extreme point within 1.5 times the interquartile range. (C) The nitrogen content of leaves decreases with increasing biomass in *Typha*-dominated (n = 24, slope_SMA_ = -8.34, r = 0.59, p = 0.0023) and removal communties (n = 6, slope_SMA_ = -9.92, r = 0.80, p = 0.0549), indicating nutrient limitation. Leaf nitrogen was not significantly correlated to aboveground biomass in *Phragmites*-dominated communities (n = 18, slope_SMA_ = -2.12, r = 0.23, p = 0.3584).

**Fig 5:**
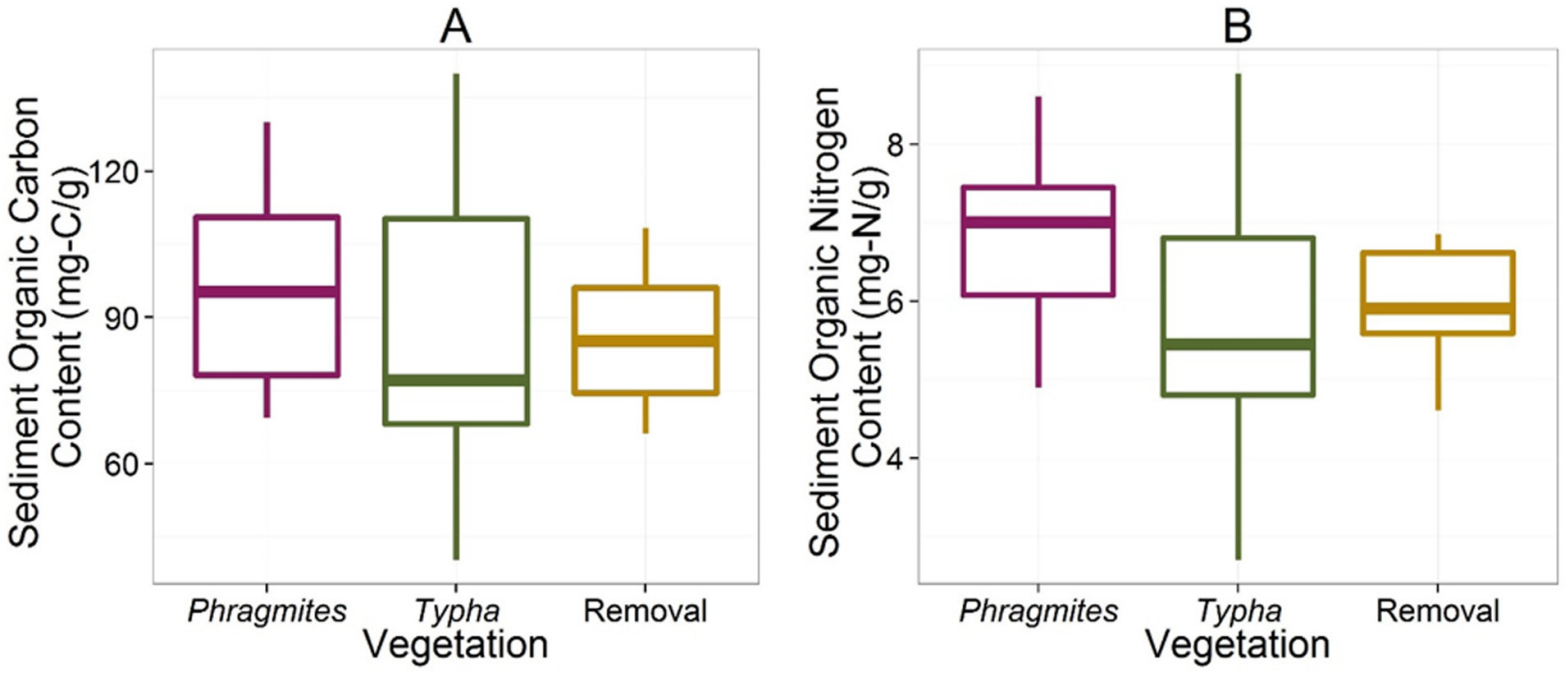
Differences in organic carbon and nitrogen in sediments among three dominant vegetation types. Sediments in *Phragmites*-dominated plant communities tend to contain (A) marginally higher organic-carbon content and (B) significantly higher organic nitrogen content than in *Typha*-dominated and removal communites. These results suggest the potential for higher organic-nitrogen storage in sediments in *Phragmites*-dominated sites. Boxplots represent the median and interquartile range, and whiskers extend to the most extreme point within 1.5 times the interquartile range.

In September 2011, one year following initial herbicide application, the removal sites in Ramshorn Marsh remained largely unvegetated, except for sparse regrowth of *Peltandra virginica* (Fig 2B). By September 2012 the removal sites had been colonized by a community dominated by *Leersia oryzoides*, *Polygonum arifolium*, *Peltandra virginica*, *Impatiens capensis*, *Scirpus fluviatilis*, and *Scirpus tabernaemontani* (Fig 2C). This community was distinct in species composition from other marsh communities dominated by *Typha angustifolia* or *Phragmites australis* (Fig 3 and Table 1). Removal communities were also characterized by lower aboveground biomass (Fig 4A and Table 2) and leaf nitrogen content (Fig 4B and Table 2), relative to *Phragmites*-dominated reference sites. In all cases, *Phragmites*-dominated sites greatly exceeded other plant communities in aboveground biomass production and leaf nitrogen content (Table 2). For both *Typha*-dominated communities and removal communities, leaf nitrogen content was negatively correlated with aboveground biomass at the plot level (p = 0.0023 and p = 0.0549, respectively). For *Phragmites*-dominated communities, this correlation was weak and of small effect (p = 0.3584, Fig 4C). Sediments in *Phragmites*-dominated sites on average contained slightly more organic carbon than *Typha*-dominated sites (Fig 5A, Table 2). Sediment organic nitrogen, however, was significantly greater for *Phragmites*-dominated sites than *Typha*-dominated sites, and slightly higher on average than in removal communities (Fig 5B and Table 2).

**Table 1:** Discriminant analysis of plant communities. Three a priori vegetation-cover types [*Phragmites*, *Typha*, Removal] were distinguished based on a weighted linear combination of component species’ biomass. (Wilks’ Λ = 0.017, Approx. F_18,26_ = 9.603, p <0.001, percent misclassified = 4.167).

Canonical Axis	Eigenvalue	% Variance Explained	Cumulative % Explained	Canonical Correlation
1	12.705	79.54	79.54	0.96
2	3.268	20.46	100.00	0.87

**Table 2:** Summary of p values from ANOVAs comparing vegetated communities before and after herbicide application to “Removal” sites in Ramshorn Marsh. Results significant at α = 0.10 are shown in bold. Results that were non-significant after correcting for multiple comparisons are marked with an asterisk. For full ANOVA tables and false-discovery corrections, see Supporting Information.

				Before-Treatment Comparisons			After-Treatment Comparisons			
Dependent Variable	Years Included in Analysis	Time	Vegetation	*Phragmites* v Removal^1^	*Typha* v Removal^2^	*Phragmites* v Typha^3^	*Phragmites* v Removal^1^	*Typha* v Removal^2^	*Phragmites* v *Typha*^3^	Time x Vegetation
Denitrification Potential	2010–2012	**0.0018**	0.1122	0.8896	0.9675	0.9269	**0.0471**	0.9134	**0.0591**	0.9066
	2011–2012	**0.0046**	0.1938				**0.0874**	0.9259	0.7594	0.2590
Sediment Ammonium	2010–2012	**0.0335**	**0.0162**	0.9858	0.9772	0.9472	**0.0007**	**0.0054**	0.4215	**0.0330**
	2011–2012	**0.0861**	**0.0016**				**0.0007**	**0.0055**	0.9244	**0.0385**
Sediment Organic Content	2010–2012	0.8467	0.4542	0.6109	0.9205	0.6935	0.2737	0.7354	0.4446	0.8830
	2011–2012	0.5938	0.4099				0.2763	0.6175	0.1108	0.7872
Aboveground Biomass	2011–2012	0.6601	**0.0079**						**0.0079**	0.9274
	Sep. 2012		0.1186				**0.0464**		0.1386	
Leaf Nitrogen	2011–2012	**0.0001**	**0.0004**						**0.0004**	0.5456
	Sep. 2012		**0.0408**				**0.0408**		*0.0651	
Sediment Carbon	2010–2012	0.3356	0.1318				0.1096		**0.0609**	0.5124
Sediment Nitrogen	2010–2012	0.3997	**0.0237**				*0.0549		**0.0077**	0.6416

^1^ Planned comparisons between removal sites in which *Phragmites* was treated with herbicide and reference sites in which *Phragmites* stands remained untreated.

^2^ Planned comparisons between removal sites in Ramshorn Marsh and *Typha*-dominated stands in Ramshorn Marsh.

^3^ Planned comparisons between *Phragmites*- and *Typha*-dominated reference sites.

### Porewater Nutrients

**Fig 6:**
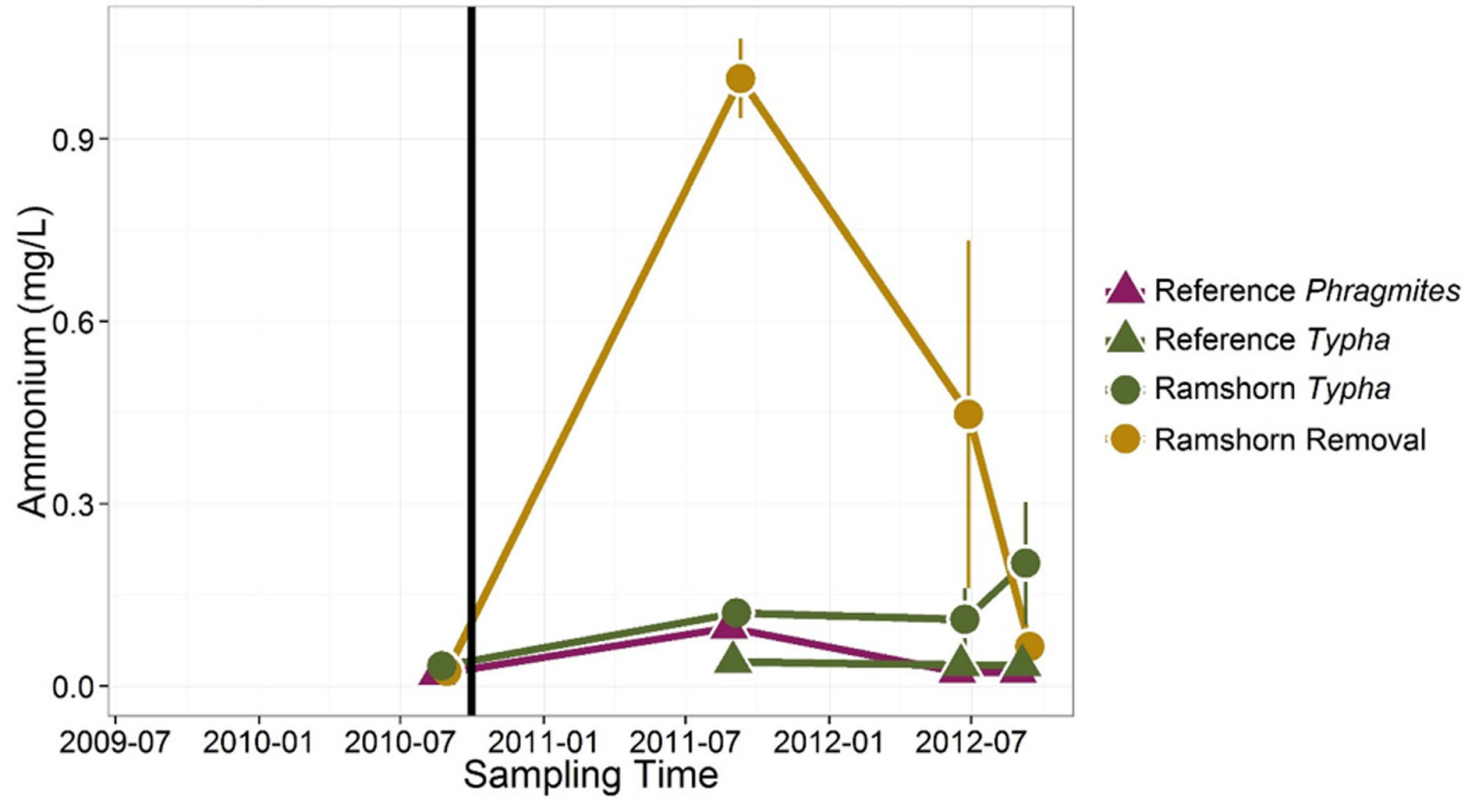
Sediment ammonium content before and after *Phragmites* removal in different vegetation types. Following herbicide treatment in September 2010 (vertical black line), ammonium concentrations in sediments of treated sites increased by an order of magnitude relative to sites vegetated with *Phragmites* or *Typha*. Ammonium concentrations returned to pretreatment levels within two years of initial treatment. Error bars show standard errors.

Average porewater nitrate concentrations remained at or below 0.02 mg/L (i.e., the detection limit for our analytical methods) across all treatments throughout our study. Therefore, only results of porewater ammonium are reported. Following initial herbicide treatment, porewater ammonium concentrations in removal sites increased by over an order of magnitude relative to all vegetated sites (Fig 6 and Table 2, p = 0.0007). They returned to pretreatment levels within two years (Fig 6).

### Denitrification Potential

**Fig 7:**
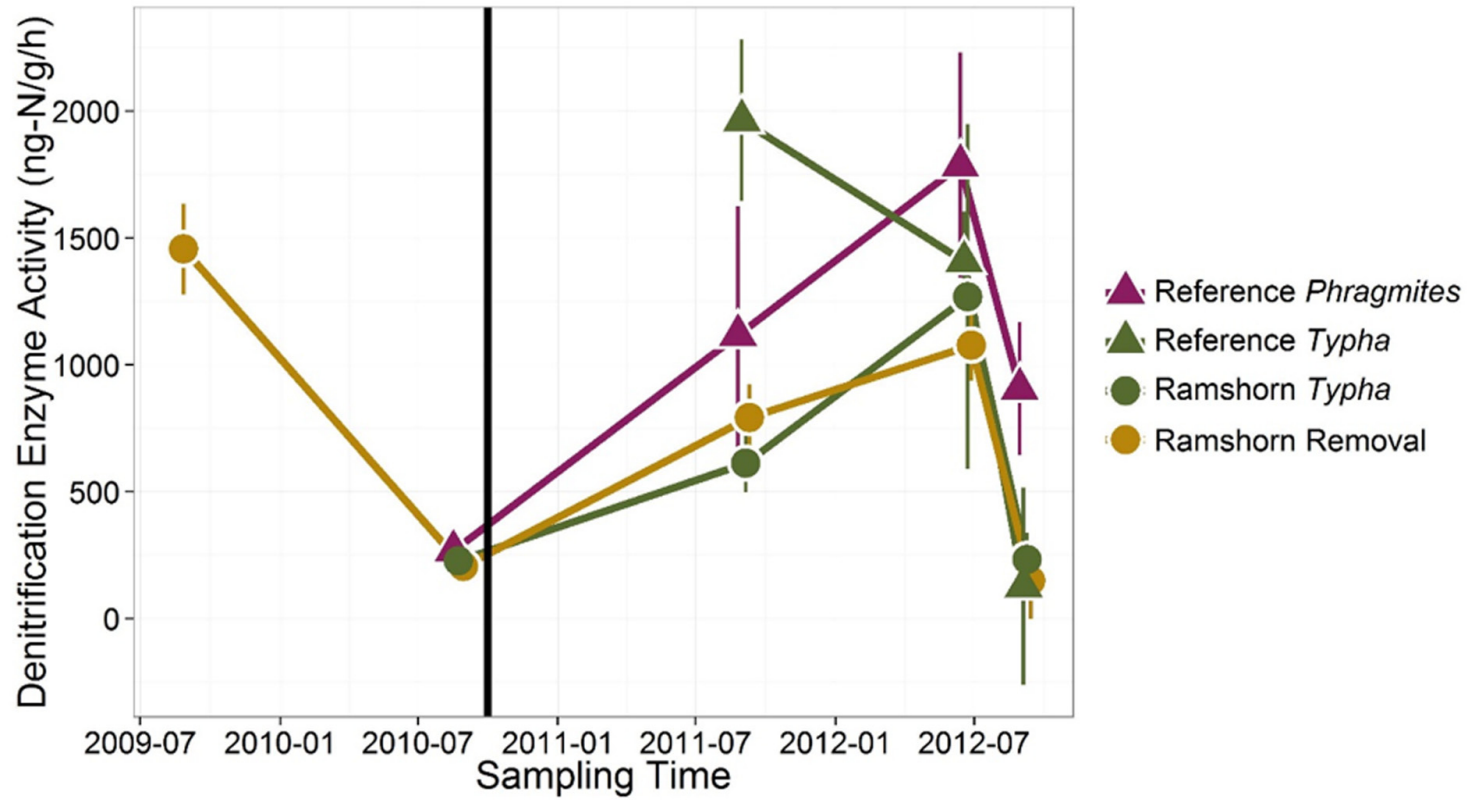
Denitrification potentials of sediments before and after *Phragmites* removal in different vegetation types. Denitrification potentials at all sites showed substantial interannual and interseasonal variability. Relative to *Phragmites*-dominated sites (purple), denitrification potentials in removal sites (brown) decreased by 50% following initial herbicide treatment (vertical black line). This effect persisted for two years following removal. Error bars show standard errors.

The most striking source of variation in denitrification potential was time, with average measurements varying nearly 8-fold among seasons and years (Fig 7 and Table 2). Nevertheless, denitrification potential in removal sites decreased significantly by approximately 50% relative to intact *Phragmites*-dominated reference locations (Fig 7 and Table 2, p = 0.0471). This effect remained remarkably constant two years following initial herbicide application (Fig 7). With the exception of anomalously high denitrification potential measurements at the reference *Typha* sites in September 2011, denitrification potentials were consistently highest in *Phragmites*-dominated sites, and were significantly higher than *Typha*-dominated sites in Ramshorn Marsh at α = 0.10 (Table 2).

## Discussion

The removal of invasive *Phragmites australis* had a significant negative impact on nitrogen-removal processes in Ramshorn Marsh, increasing sediment ammonium concentrations by an order of magnitude and reducing the ability of the sediment microbial community to remove nitrogen by 50% relative to *Phragmites*-dominated marshes. The impacts we observed in this study are consistent with the results of the only other study to examine changes in denitrification potential following eradication of *Phragmites* [30]. Although it is metabolically impossible for glyphosate itself to have a direct negative effect on prokaryotes, it is possible that the surfactant or other proprietary ingredients added with the herbicide may have affected the sediment microbial community [46]. However, glyphosate and LI 700 surfactants have been demonstrated to have negligible effects on organisms other than plants and do not persist in biologically available form in aquatic environments, particularly in well-flushed tidal environments such as the one examined in this study [46]. Notably, our first post-removal measurements were conducted a full year after initial herbicide application. Due to this time lag and the considerable tidal flushing that occurs in Ramshorn Marsh, it is very unlikely that the herbicide itself could have persisted long enough to produce the effects we observed.

The effectiveness of *Phragmites* in promoting nitrogen-removal services, relative to native plant communities in our sites, is consistent with what one would expect based on extensive characterization of this plant invader [22, 31, 47]. *Phragmites* is known to sequester larger pools of nitrogen in both above and belowground growth [22], as well as to aerate otherwise anoxic sediments [26], promoting removal of nitrogen via coupled nitrification-denitrification. Following herbicide treatment, assimilation of inorganic nitrogen into plant tissues would cease, and roots would no longer introduce oxygen to sediments, hindering oxidation of ammonium to nitrate. This sequence of events should result in a build-up of sediment ammonium, which we clearly observed (Fig 6). Though total inorganic nitrogen levels increased, we expected denitrification potential to decrease due to limitation of denitrifying microbes by nitrate (oxidant). Not only did we observe an initial decrease in denitrification potential in treated sites relative to intact *Phragmites*-dominated reference sites, this effect persisted two years following the initial treatment, even as native plant communities began to recolonize the treated sites.

This study is one of the few that have characterized the plant community that recolonized sites following *Phragmites* eradication [29]. Consistent with those studies, we found that, rather than recovering to a similar native plant community found elsewhere in the marsh, the sites shifted to a completely different suite of species. The results of our vegetation surveys were consistent with surveys of 1.0 m^2^ plots conducted by the Nature Conservancy at the same marsh locations [32]. In this case, the new community was characterized primarily by the presence of *Leersia oryzoides* and *Polygonum arifolium*, rather than *Typha*-dominated marshes that are typical of emergent wetlands in the Hudson River (Fig 3). Notably, many of these species are commonly found in the understory of *Typha*-dominated marshes in the Hudson River, and may represent an early stage in the succession of a disturbed tidal marsh community in this system [48]. This new community attained only 1/4 of the peak biomass of a *Phragmites*-dominated plant community and contained less than half the nitrogen content of a mature *Phragmites* leaf (Fig 4A and 4B). Overall, the capacity of this community to store nitrogen in aboveground tissue is severely reduced relative to both *Phragmites*- and *Typha*-dominated communities (Fig 4). The net effect of species succession in this case could serve to increase the amount of time needed for a site to recover nitrogen-removal capacity following invasive species removal. Though we did not measure belowground traits of the plant communities, the lag in recovery of denitrification potential (Fig 7) suggests that plant communities may also differ in their belowground effects on microbial processes. In order to fully understand impacts of plant-community alterations on ecosystem processes, belowground measurements should be given greater attention in future studies.

The success of *Phragmites* as an invader is often attributed to its ability to alter sediment nutrient cycles such that it has a competitive advantage relative to native plant species [22]. Specifically, *Phragmites* has been shown to increase pools of organic nitrogen relative to inorganic nitrogen in sediments, which other native plants are less efficient at assimilating [22, 49]. Our results suggest that such a mechanism may be at work in the Hudson River. We found significantly higher concentrations of total organic nitrogen in sediments of *Phragmites*-dominated sites, relative to sites inhabited by native *Typha*-dominated plant communities (Fig 4B and Table 2). Moreover, total aboveground biomass was negatively correlated with leaf nitrogen in native plant communities, which could be interpreted as a sign of nutrient competition within a plant community (Fig 3C). This correlation was much weaker for *Phragmites*, which is consistent with relaxed nutrient competition in *Phragmites*-dominated sites due to access to an additional pool of organic nitrogen. Relaxed nutrient competition, along with re-sprouting from rhizomes and quickly producing high aboveground biomass, may explain the difficulty in permanently eradicating stands of *Phragmites* [29].

Overall, *Phragmites*-dominated plant communities assimilate more nitrogen in aboveground biomass (Fig 3) and store more nitrogen in organic form in sediments (Fig 4) than native plant communities. Further, the removal of *Phragmites* resulted in significant increases in dissolved inorganic nitrogen pools (Fig 6) and significant decreases in the potential of the microbial community to permanently remove nitrogen to the atmosphere via denitrification (Fig 7). Together, these results suggest a management trade-off between eradicating *Phragmites* to restore a diverse native plant community and promoting nitrogen-removal ecosystem services in the wetland. Certainly, management actions such as seasonal timing of treatment or immediate replanting of native plants could help ameliorate these impacts, but follow-up herbicide treatments are always required, and some degree of disturbance is likely to be inevitable [50, 51]. In light of this trade-off in management priorities, the question then becomes: When and where is *Phragmites* eradication likely to significantly impact local water quality? For small, isolated patches of *Phragmites* in a system like the Hudson, which is relatively hydrologically open and well flushed by periodic tidal inundation, the impact on water quality is likely to be quite small relative to the benefit of restoring native diversity and improving habitat conditions for wildlife. However, for systems in which *Phragmites* has become the major plant community in the landscape, complete eradication is not only unlikely [50] but may result in water quality problems that outweigh the benefits of biodiversity restoration, particularly for aquatic systems where water sources are smaller or hydrologically isolated. Invasive-species management highlights the need to develop specific management priorities for ecosystems. Frameworks are continually being developed that seek to simultaneously optimize multiple ecosystem services, with the understanding that management priorities may in some cases conflict [52–54].

*Phragmites* management in the Hudson River Estuary provides one example of a situation in which biodiversity does not maximize delivery of an ecosystem service. In this case a dominant species with traits that maximize opportunistic nutrient acquisition and high biomass production also maximizes nitrogen-removal ecosystem services. In addition to nitrogen removal, *Phragmites* is credited with additional ecosystem services including carbon and heavy metal sequestration, soil stabilization, and habitat provisioning for both common and rare native animals [19]. Other exotic grasses have been found to store carbon and provide forage for grazing animals [55]. Ecosystems dominated by invasive species and low-diversity communities challenge simple assumptions that biodiversity should beget greater value for multiple ecosystem functions. Particularly in urbanized and highly modified systems, invasive species like *Phragmites* can have quantifiable functional value both to the environment and local human societies that may not be easily achieved with native communities. In such cases, a holistic approach that begins with explicit management priorities, and considers potential trade-offs among management outcomes, is likely to provide the best solutions to management and restoration.

## Conclusions

The removal of invasive *Phragmites australis* from tidal freshwater marshes of the Hudson River resulted in a significant short-term increase in sediment ammonium concentrations, and a significant loss in denitrification potential that persisted for at least two years. These changes suggest the potential for an important trade-off between conserving native plant diversity and maintaining nitrogen-removal ecosystem services in wetland ecosystems. Therefore, management approaches that incorporate a balanced assessment of costs associated with keeping versus removing invasive plants are likely to provide the best outcomes for maintaining both biodiversity and water quality.

## Supporting Information

S1 File Statistical Output and Code. (PDF)
