# Supplementary material for: Effects of Invasive-Plant Management on Nitrogen-Removal Services in Freshwater Tidal Marshes
Source: PLoS One. 2016 Feb 25;11(2):e0149813. doi: 10.1371/journal.pone.0149813 (PMC4767409; doi:10.1371/journal.pone.0149813)
Supplement: S1 File — (PDF) [file pone.0149813.s001.pdf]

Supporting Information

Output from JMP

Table S1: Summary of one-way ANOVAs of plant traits collected in September 2012 for three vegetation cover types [*Phragmites*, *Typha*, Removal], two-way ANOVAs of plant traits collected September 2011-September 2012 for *Phragmites* and *Typha* communities only, and two-way ANOVAs of sediment carbon and nitrogen content collected August 2010-September 2012. Leaf nitrogen content in September 2012 was calculated as an average of the component species of the community, weighted by their biomass. Effects significant at α = 0.05 are shown in bold. Analyses performed in JMP.

Source	df	Sum of Squares	F Ratio	p
Aboveground Biomass, September 2012				
Vegetation	2	35.12	2.73	0.1186
**Contrast: *Phragmites* v. Removal**	1	34.30	5.33	**0.0464**
Contrast: *Phragmites* v. *Typha*	1	17.00	2.64	0.1386
Error	9	57.94		
Total	11	93.07		
Aboveground Biomass, 2011-2012, Reference *Phragmites* and *Typha* only				
Time	2	4.13	0.42	0.6601
**Vegetation**	1	45.49	9.39	**0.0079**
Time x Vegetation	2	0.73	0.07	0.9274
Error	15	72.64		
Total	20	121.56		
Leaf Nitrogen Content, September 2012				
**Vegetation**	2	117.67	4.66	**0.0408**
**Contrast: *Phragmites* v. Removal**	1	115.28	9.13	**0.0144**
Contrast: *Phragmites* v. *Typha*	1	55.65	4.41	0.0651
Error	9	113.58		
Total	11	231.25		
Leaf Nitrogen Content, 2011-2012, Reference *Phragmites* and *Typha* only				
**Time**	2	459.31	19.80	**<0.0001**
**Vegetation**	1	241.12	20.78	**0.0004**
Time x Vegetation	2	14.64	0.63	0.5456
Error	15	173.99		
Total	20	1001.32		
Sediment Carbon Content, August 2010-September 2012				
Time	3	1576.98	1.17	0.3356
Vegetation	2	1931.61	2.14	0.1318
Contrast: *Phragmites* v. Removal	1	1211.47	2.69	0.1096
Contrast: *Phragmites* v. *Typha*	1	1685.38	3.74	0.0609
Time x Vegetation	6	2404.57	0.89	0.5124
Error	36	16208.37		

Total	47	21914.03		
Sediment Nitrogen Content, August 2010-September 2012				
Time	3	4.4462	1.01	0.3997
**Vegetation**	2	12.2090	4.16	**0.0237**
Contrast: *Phragmites* v. Removal	1	5.7760	3.94	0.0549
**Contrast: *Phragmites* v. *Typha***	1	11.6806	7.96	**0.0077**
Time x Vegetation	6	6.2764	0.71	0.6416
Error	36	52.8383		
Total	47	73.6617		

Table S2: Summary of two-way ANOVAs comparing denitrification potential and sediment properties of three vegetation treatments [Ramshorn-Removal, Ramshorn-*Typha*, Reference-*Phragmites*] before [August 2010] and after [September 2011, June 2012, September 2012] herbicide application. Effects significant at α = 0.05 are shown in bold. Analyses performed in JMP.

**Source**	**df**	**Sum of Squares**	**F Ratio**	**P**
Denitrification Potential, 2010-2012				
**Time**	3	7.32×10^{6}	6.57	**0.0018**
Vegetation	2	1.76×10^{6}	2.37	0.1122
Time x Vegetation	6	7.68×10^{5}	0.34	0.9066
Contrast: *Phragmites* v Removal 2010	1	7.29×10^{3}	0.02	0.8896
Contrast: *Typha* v Removal 2010	1	6.38×10^{2}	0.00	0.9675
Contrast: *Phragmites* v *Typha* 2010	1	3.19×10^{3}	0.01	0.9269
**Contrast: *Phragmites* v Removal 2011-2012**	1	1.61×10^{6}	4.33	**0.0471**
Contrast: *Typha* v Removal 2011-2012	1	4.47×10^{3}	0.01	0.9134
Contrast: *Phragmites* v *Typha* 2011-2012	1	1.44 x 106	3.88	0.0591
Error	27	1.00×10^{7}		
Total	38	1.97×10^{7}		
Ammonium, 2010-2012				
**Time**	3	0.6398	3.42	**0.0335**
**Vegetation**	2	0.6145	4.92	**0.0162**
**Time x Vegetation**	6	1.0480	2.79	**0.0330**
Contrast: *Phragmites* v Removal 2010	1	0.0000	0.00	0.9858
Contrast: *Typha* v Removal 2010	1	0.0000	0.00	0.9772
Contrast: *Phragmites* v *Typha* 2010	1	0.0003	0.00	0.9472
**Contrast: *Phragmites* v Removal 2011-2012**	1	0.9370	15.00	**0.0007**
**Contrast: *Typha* v Removal 2011-2012**	1	0.5831	9.34	**0.0054**
Contrast: *Phragmites* v *Typha* 2011-2012	1	0.0418	0.67	0.4215
Error	24	1.4987		
Total	35	4.1843		
Organic Content, 2010-2012				
Time	3	1.44×10^{-3}	0.26	0.8467
Vegetation	2	2.89×10^{-3}	0.81	0.4542
Time x Vegetation	6	4.01×10^{-3}	0.38	0.8830
Contrast: *Phragmites* v Removal 2010	1	4.71×10^{-4}	0.26	0.6109

Contrast: *Typha* v Removal 2010	1	1.80×10^{-5}	0.01	0.9205
Contrast: *Phragmites* v *Typha* 2010	1	2.82×10^{-4}	0.16	0.6935
Contrast: *Phragmites* v Removal 2011-2012	1	2.22×10^{-3}	1.25	0.2737
Contrast: *Typha* v Removal 2011-2012	1	2.07×10^{-4}	0.12	0.7354
Contrast: *Phragmites* v *Typha* 2011-2012	1	1.07×10^{-3}	0.60	0.4446
Error	27	4.79×10^{-2}		
Total	38	5.67×10^{-2}		

Table S3: Summary of two-way ANOVAs comparing denitrification potential and sediment properties of four vegetation treatments [Ramshorn-Removal, Ramshorn-*Typha*, Reference-*Typha*, Reference-*Phragmites*] only after [September 2011, June 2012, September 2012] herbicide application. Effects significant at α = 0.05 are shown in bold. Analyses performed in JMP.

**Source**	**df**	**Sum of Squares**	**F Ratio**	**p**
Denitrification Potential, 2011-2012				
**Time**	2	6.88×10^{6}	6.80	**0.0046**
Vegetation	3	2.58×10^{6}	1.70	0.1938
Contrast: Reference-*Phragmites* v Ramshorn-Removal	1	1.61×10^{6}	3.18	0.0874
Contrast: Ramshorn-*Typha* v Ramshorn-Removal	1	4.47×10^{3}	0.01	0.9259
Contrast: Reference-*Phragmites* v Reference-*Typha*	1	4.85×10^{4}	0.10	0.7594
Time x Vegetation	6	2.71×10^{6}	0.89	0.2590
Error	27	1.21×10^{7}		
Total	38	2.43×10^{7}		
Ammonium, 2011-2012				
Time	2	0.3402	2.72	0.0861
**Vegetation**	3	1.2993	6.93	**0.0016**
**Contrast: Reference-*Phragmites* v Ramshorn-Removal**	1	0.9370	14.99	**0.0007**
**Contrast: Ramshorn-*Typha* v Ramshorn-Removal**	1	0.5831	9.33	**0.0055**
Contrast: Reference-*Phragmites* v Reference-*Typha*	1	0.0092	0.01	0.9244
**Time x Vegetation**	6	1.0091	2.69	**0.0385**
Error	24	1.5005		
Total	35	4.1490		
Organic Content, 2010-2012				
Time	2	4.60×10^{-4}	0.53	0.5938
Vegetation	3	1.30×10^{-3}	1.00	0.4099
Contrast: Reference-*Phragmites* v Ramshorn-Removal	1	5.37×10^{-4}	1.24	0.2763
Contrast: Ramshorn-*Typha* v Ramshorn-Removal	1	1.11×10^{-4}	0.26	0.6175
Contrast: Reference-*Phragmites* v Reference-*Typha*	1	1.19×10^{-3}	2.74	0.1108
Time x Vegetation	6	1.35×10^{-3}	0.52	0.7872
Error	27	1.04×10^{-2}		
Total	38	1.35×10^{-2}		

False-Discovery-Rate (FDR) Corrections for Multiple Comparisons

Procedure for Benjamini and Hochberg (1995) FDR Corrections:

1) Rank multiple comparison p-values from highest to lowest.
2) Compute corrected critical α' = i/k * α, where i refers to the ith observation, k is the total number of compariosns, and α is the desired experiment-wise error rate (here 0.10).
3) The first p_i for which $p_i < \alpha'$ is the critical value for the experiment.

i	α'	**Denitrification (2010-2012)**	**Ammonium (2010-2012)**
1	0.0167	0.9675	0.9858
2	0.0333	0.9269	0.9772
3	0.0500	0.9134	0.9472
4	0.0667	0.8896	0.4215
5	0.0833	0.0591	0.0054
6	0.1000	0.0471	0.0007
**Critical value**		**0.0591**	**0.0054**

i	α'	**Denitrification (2011-2012)**	**Ammonium (2011-2012)**
1	0.0333	0.9259	0.9244
2	0.0667	0.7594	0.0055
3	0.1000	0.0874	0.0007
**Critical value**		**0.0874**	**0.0055**

i	α'	**AG Biomass (Sep.2012)**	**Leaf N (Sep.2012)**	**Sediment C (Sep.2012)**	**Sediment N (Sep.2012)**
1	0.0500	0.1386	0.0651*	0.1096	0.0549*
2	0.1000	0.0464	0.0408	0.0609	0.0077
**Critical value**		**0.0464**	**0.0408**	**0.0609**	**0.0077**

Output from R

Packages

```
library(ggplot2)
library(bear)
```

Data and Formatting

```
setwd(filepath5)
hudson <- read.csv("20141022_Sediment_AllYears.csv")
treatmentmeans <- read.csv("20140428_Sediment_AllYears_Treatmentmeans.csv")
# Convert 'Time' from factor to date
hudson$Time <- as.Date(hudson$Time)
# Convert 'Vegetation' from factor to ordered factor
hudson$Vegetation <- ordered(hudson$Vegetation, levels = c("Phragmites", "Typha",
"Removal"))
# Convert 'Time' from factor to date
treatmentmeans$Time <- as.Date(treatmentmeans$Time)
# Convert 'Vegetation' from factor to ordered factor
treatmentmeans$Vegetation <- ordered(treatmentmeans$Vegetation, levels = c("Phragmites",
    "Typha", "Removal"))
# Convert 'Site_Treatment' from factor to ordered factor
treatmentmeans$Site_Treatment <- ordered(treatmentmeans$Site_Treatment, levels =
c("Reference Phragmites", "Reference Typha", "Ramshorn Typha", "Ramshorn Removal"))
# Change base text size for figures
theme_set(theme_bw(base_size = 20))
```

Plot Biomass by Vegetation Type

```
# Create a summary table for Biomass data at the plot level using summarySE{bear}
Biomass <- summarySE(hudson, measurevar = "AGBiomass_kg.m2", groupvar = c("Time",
    "ReplicateCode", "Vegetation"), na.rm = T)
# Create a boxpolot for biomass
biomass <- ggplot(Biomass, aes(x = Vegetation, y = AGBiomass_kg.m2, color = Vegetation))
biomass + geom_boxplot(size = 1.5) + scale_color_manual(values = c("maroon4",
"darkolivegreen", "darkgoldenrod")) + ylab(expression(paste("Biomass (kg/", m^{2}, ")"))) +
theme(legend.position = "none") + theme(axis.text.x = element_text(face = c("italic", "italic",
"plain"), color = "black")) + theme(axis.text.y = element_text(color = "black")) + ggtitle("A")
```

Plot Leaf N Content by Vegetation Type

```
LeafN <- summarySE(hudson, measurevar = "PlantN_mg.gDW", groupvar = c("Time",
    "ReplicateCode", "Vegetation"), na.rm = T)
LeafNcontent <- ggplot(LeafN, aes(x = Vegetation, y = PlantN_mg.gDW, color = Vegetation))
LeafNcontent + geom_boxplot(size = 1.5) + scale_color_manual(values = c("maroon4",
"darkolivegreen", "darkgoldenrod")) + ylab("Leaf Nitrogen Content (mg-N/g)") +
theme(legend.position = "none") + theme(axis.text.x = element_text(face = c("italic", "italic",
"plain"), color = "black")) + theme(axis.text.y = element_text(color = "black")) + ggtitle("B")
```

Graph aboveground biomass v Leaf nitrogen content

A negative slope would indicate nutrient competition

```
Biomass_LeafN_means <- cbind(Biomass, LeafN)
Biomass_LeafN <- ggplot(Biomass_LeafN_means, aes(x = AGBiomass_kg.m2, y =
PlantN_mg.gDW, color = Vegetation))
Biomass_LeafN + geom_abline(intercept = 42.61210463, slope = -2.120325886, color =
"maroon4",     size = 2) + geom_abline(intercept = 42.14073928, slope = -8.3415148, color =
"darkolivegreen", size = 2) + geom_abline(intercept = 23.3532942, slope = -9.917019514, color
= "darkgoldenrod", size = 2) + geom_point(size = 5, color = "white") + geom_point(size = 4) +
scale_color_manual(values = c("maroon4", "darkolivegreen", "darkgoldenrod"), labels =
expression(paste(italic("Phragmites")), paste(italic("Typha")), paste("Removal"))) +
xlab(expression(paste("Biomass (kg/", m^{2}, ")"))) + ylab("Leaf Nitrogen Content (mg-N/g)")
+ theme(legend.title = element_blank(), legend.key = element_blank(), legend.text.align = 0) +
ggtitle("C") + scale_y_continuous(limits = c(10, 40))
```

Create Plot for Sediment C content

```
SedC <- summarySE(hudson, measurevar = "SedCmg.gDW", groupvar = c("Time",
"ReplicateCode", "Vegetation"), na.rm = T)
SedCcontent <- ggplot(SedC, aes(x = Vegetation, y = SedCmg.gDW, color = Vegetation))
SedCcontent + geom_boxplot(size = 1.5) + scale_color_manual(values = c("maroon4",
"darkolivegreen", "darkgoldenrod")) + ylab("Sediment Organic Carbon \nContent (mg-N/g)") +
theme(legend.position = "none") + theme(axis.text.x = element_text(face = c("italic", "italic",
"plain"), color = "black")) + theme(axis.text.y = element_text(color = "black")) + ggtitle("A")
```

Create Plot for Sediment N content

```
SedN <- summarySE(hudson, measurevar = "SedNmg.gDW", groupvar = c("Time",
"ReplicateCode", "Vegetation"), na.rm = T)
SedNcontent <- ggplot(SedN, aes(x = Vegetation, y = SedNmg.gDW, color = Vegetation))
SedNcontent + geom_boxplot(size = 1.5) + scale_color_manual(values = c("maroon4",
"darkolivegreen", "darkgoldenrod")) + ylab("Sediment Organic Nitrogen \nContent (mg-N/g)")
+ theme(legend.position = "none") + theme(axis.text.x = element_text(face = c("italic", "italic",
"plain"), color = "black")) + theme(axis.text.y = element_text(color = "black")) + ggtitle("B")
```

Plot Sediment Ammonium over Time

```
# Create an object that refers to my ammonium variable
NH4 <- treatmentmeans$Mean.NH4_mgL.
# Create an object that refers to standard errors for y error bars
NH4se <- treatmentmeans$Std.Err.NH4_mgL.
# Create a position_dodge to keep treatment points and errorbars from overlapping
pd <- position_dodge(width = 20)
# Create an object indicating the date when herbicide application was performed (for v line)
herbicide <- as.Date("2010-09-15")
# Create an object that indicates whether sites were reference or treatment sites (for ease of
reading legend title)
Site_Treatment <- treatmentmeans$Site_Treatment
```

```
# Create plot
NH4Time <- ggplot(treatmentmeans, aes(x = Time, y = NH4, color = Site_Treatment,
    shape = Site_Treatment))
NH4Time + geom_line(size = 2, position = pd, aes(color = Site_Treatment)) +
geom_errorbar(aes(ymin = NH4 - NH4se, ymax = NH4 + NH4se, width = 0), position = pd,
size = 1) + geom_point(size = 9, position = pd, color = "white") + geom_point(size = 7, position
= pd) + geom_vline(xintercept = as.numeric(as.Date("2010-09-30", format = "%Y-%m-%d")),
size = 2) + scale_color_manual(name = "Site and Vegetation", values = c("maroon4",
"darkolivegreen", "darkolivegreen", "darkgoldenrod"),   labels = expression(paste("Reference ",
italic("Phragmites")), paste("Reference ", italic("Typha")), paste("Ramshorn ",
italic("Typha")), paste("Ramshorn Removal")), guide = guide_legend(title = NULL)) +
scale_shape_manual(values = c(17, 17, 19, 19), name = "Site and Vegetation", labels =
expression(paste("Reference ", italic("Phragmites")), paste("Reference ", italic("Typha")),
paste("Ramshorn ", italic("Typha")), paste("Ramshorn Removal")), guide = guide_legend(title
= NULL)) + theme(legend.key = element_blank(), legend.text.align = 0) + ylab("Ammonium
(mg/L)") + xlab("Sampling Time") + theme(axis.text.x = element_text(color = "black")) +
theme(axis.text.y = element_text(color = "black"))
```

Plot Denitrification Rates over Time

```
# Create an object that refers to my dentirification variable
DEA <- treatmentmeans$Mean.DEA..ng.N.g.hr..
# Create an object that refers to standard errors for y error bars
DEAse <- treatmentmeans$Std.Err.DEA..ng.N.g.hr..

# Create plot
DenitTime <- ggplot(treatmentmeans, aes(x = Time, y = DEA, color = Site_Treatment,
    shape = Site_Treatment))
DenitTime + geom_line(size = 2, position = pd, aes(color = Site_Treatment)) +
geom_errorbar(aes(ymin = DEA - DEAse, ymax = DEA + DEAse, width = 0), position = pd,
size = 1) + geom_point(size = 9, position = pd, color = "white") + geom_point(size = 7, position
= pd) + geom_vline(xintercept = as.numeric(as.Date("2010-09-30", format = "%Y-%m-%d")),
size = 2) + scale_color_manual(name = "Site and Vegetation", values = c("maroon4",
"darkolivegreen", "darkolivegreen", "darkgoldenrod"), labels = expression(paste("Reference ",
italic("Phragmites")), paste("Reference ", italic("Typha")), paste("Ramshorn ",
italic("Typha")), paste("Ramshorn Removal")), guide = guide_legend(title = NULL)) +
scale_shape_manual(values = c(17, 17, 19, 19), name = "Site and Vegetation", labels =
expression(paste("Reference ", italic("Phragmites")), paste("Reference ", italic("Typha")),
paste("Ramshorn ", italic("Typha")), paste("Ramshorn Removal")), guide = guide_legend(title
= NULL)) + theme(legend.key = element_blank(), legend.text.align = 0) +
ylab("Denitrification Enzyme Activity (ng-N/g/h)") + xlab("Sampling Time") +
theme(axis.text.x = element_text(color = "black")) + theme(axis.text.y = element_text(color =
"black"))
```
